# Pilus of *Streptococcus pneumoniae*: structure, function and vaccine potential

**DOI:** 10.3389/fcimb.2023.1270848

**Published:** 2023-09-20

**Authors:** Chenglin Miao, Yali Cui, Ziyi Yan, Yongmei Jiang

**Affiliations:** ^1^ Department of Laboratory Medicine, West China Second University Hospital, Sichuan University, Chengdu, Sichuan, China; ^2^ Department of Laboratory Medicine, Meishan Women and Children’s Hospital, Alliance Hospital of West China Second University Hospital, Sichuan University, Meishan, Sichuan, China; ^3^ Department of Laboratory Medicine, West China Second University Hospital (Tianfu), Sichuan University/Sichuan Provincial Children’s Hospital, Meishan, Sichuan, China; ^4^ Key Laboratory of Birth Defects and Related Diseases of Women and Children (Sichuan University), Ministry of Education, Chengdu, Sichuan, China

**Keywords:** *Streptococcus pneumoniae*, pilus, structure, pilin, protein vaccine

## Abstract

The pilus is an extracellular structural part that can be detected in some *Streptococcus pneumoniae* (*S. pneumoniae*) isolates (type I pili are found in approximately 30% of strains, while type II pili are found in approximately 20%). It is anchored to the cell wall by LPXTG-like motifs on the peptidoglycan. Two kinds of pili have been discovered, namely, pilus-1 and pilus-2. The former is encoded by pilus islet 1 (PI-1) and is a polymer formed by the protein subunits RrgA, RrgB and RrgC. The latter is encoded by pilus islet 2 (PI-2) and is a polymer composed mainly of the structural protein PitB. Although pili are not necessary for the survival of *S. pneumoniae*, they serve as the structural basis and as virulence factors that mediate the adhesion of bacteria to host cells and play a direct role in promoting the adhesion, colonization and pathogenesis of *S. pneumoniae*. In addition, as candidate antigens for protein vaccines, pili have promising potential for use in vaccines with combined immunization strategies. Given the current understanding of the pili of *S. pneumoniae* regarding the genes, proteins, structure, biological function and epidemiological relationship with serotypes, combined with the immunoprotective efficacy of pilins as protein candidates for vaccines, we here systematically describe the research status and prospects of *S. pneumoniae* pili and provide new ideas for subsequent vaccine research and development.

## 1 Introduction

*Streptococcus pneumoniae* (*S. pneumoniae*), a common bacterium colonizing the human upper respiratory tract, is a gram-positive bacterium capable of establishing symbiotic relationships that can often lead to respiratory tract infections. After escaping the immune defense of the host, *S. pneumoniae* can cause local or systemic infections such as pneumonia, otitis media, meningitis, sepsis, nasosinusitis, bronchitis, abscesses, conjunctivitis, pericarditis, and arthritis, with high morbidity and mortality in children younger than 5 years and elderly people (O’Brien et al., 2009; Deloria Knoll et al., 2014). The WHO ranks pneumonia as the leading cause of death in children under 5 years of age (Bryce et al., 2005), and more than 50% of cases of severe pneumonia in children are caused by *S. pneumoniae*, accounting for a higher proportion of deaths than pneumonia caused by other factors (Wardlaw et al., 2006; Modell et al., 2012).

*S. pneumoniae* pili are long multisubunit structures that enhance the interaction between the pneumococcus and the host (Dzaraly et al., 2020). *S. pneumoniae* has a variety of virulence factors, including pili. As important candidate factors for *S. pneumoniae* protein vaccines, pili can facilitate the adherence of bacteria to the surface of host cells, recognize the extracellular matrix, and participate in biofilm formation. They also participate in invasion, which can induce an inflammatory response (Barocchi et al., 2006), and they play an important role in the processes of host tissue colonization and pathogenesis (Walker et al., 2014; Engholm et al., 2017). Additionally, anti-pilus antibodies have been observed in the serum of patients with *S. pneumoniae* infection (Ahmed et al., 2014). Research on *S. pneumoniae* pilin has yielded interesting findings, but the related reports have been relatively scattered and have lacked systematization. This review focuses on the recent findings related to the typing, structural characteristics and functions of *S. pneumoniae* pilins in addition to the immunoprotective efficacy of pilins as protein antigens to provide direction for follow-up research.

## 2 Characteristics and structure of the pilus

Similar to those of other gram-positive bacteria, the pili of *S. pneumoniae* are polymers formed by the covalent assembly of multiple pilin subunits, each of which contains a sortase (Srt)-specific LPXTG-like motif. Catalyzed by a specific sortase, protein subunits covalently polymerize, and the pilus anchor to the peptidoglycan on the surface of the cell wall (Naziga and Wereszczynski, 2017).

The genes encoding the pili of *S. pneumoniae* exist as islets. Two types of *S. pneumoniae* pili have been found, with the structure of the pilus islets and protein subunits differentiating the type I pilus (pilus-1) from the type II pilus (pilus-2). The former, encoded by pilus islet 1 (PI-1), is a polymer formed by the protein subunits RrgA, RrgB and RrgC. Pilus-1 was first described in 2006; it is critical in promoting virulence (Barocchi et al., 2006) and is expressed in approximately 20%-30% of *S. pneumoniae* strains (Moschioni et al., 2008). Pilus-2 is encoded by pilus islet-2 (PI-2) and is a polymer of the protein subunit PitB (Shaik et al., 2015). Because pilus islets have characteristics of mobile genetic elements, *S. pneumoniae* strains can express two types of pili, only one type of pilus or no pilus.

### 2.1 Characteristics of pilus-1

Pilus-1 was the first pilus found in *S. pneumoniae*, and it is also the most widely expressed type, with a prevalence of approximately 30% (Gholamhosseini-Moghaddam et al., 2015; Mayanskiy et al., 2017). Pilus-1 was first identified in the TIGR4 strain (Barocchi et al., 2006). PI-1 encodes pilus-1 in *S. pneumoniae* and includes a total of 7 genes, namely, three sortase-encoding genes, i.e., *srtC-1, srtC-2*, and *srtC-3* (formerly known as *srtB, srtC*, and *srtD*) (Iovino et al., 2020); a positive transcriptional regulator gene, i.e., *rlrA*; and three surface structural protein-encoding genes, i.e., *rrgA, rrgB* and *rrgC*. Notably, *rrgA, rrgB*, and *rrgC* encode LPXTG-like motifs (*rrgA:* YPRTG*, rrgB:* IPQTG*, rrgC:* VPDTG) for covalent polymerization between catalytic subunits recognized by the sortase that cross-links individual pilus subunits (pilus monomers) and for anchoring pilins to the cell wall surface (Dramsi et al., 2005).

**Figure 1:**
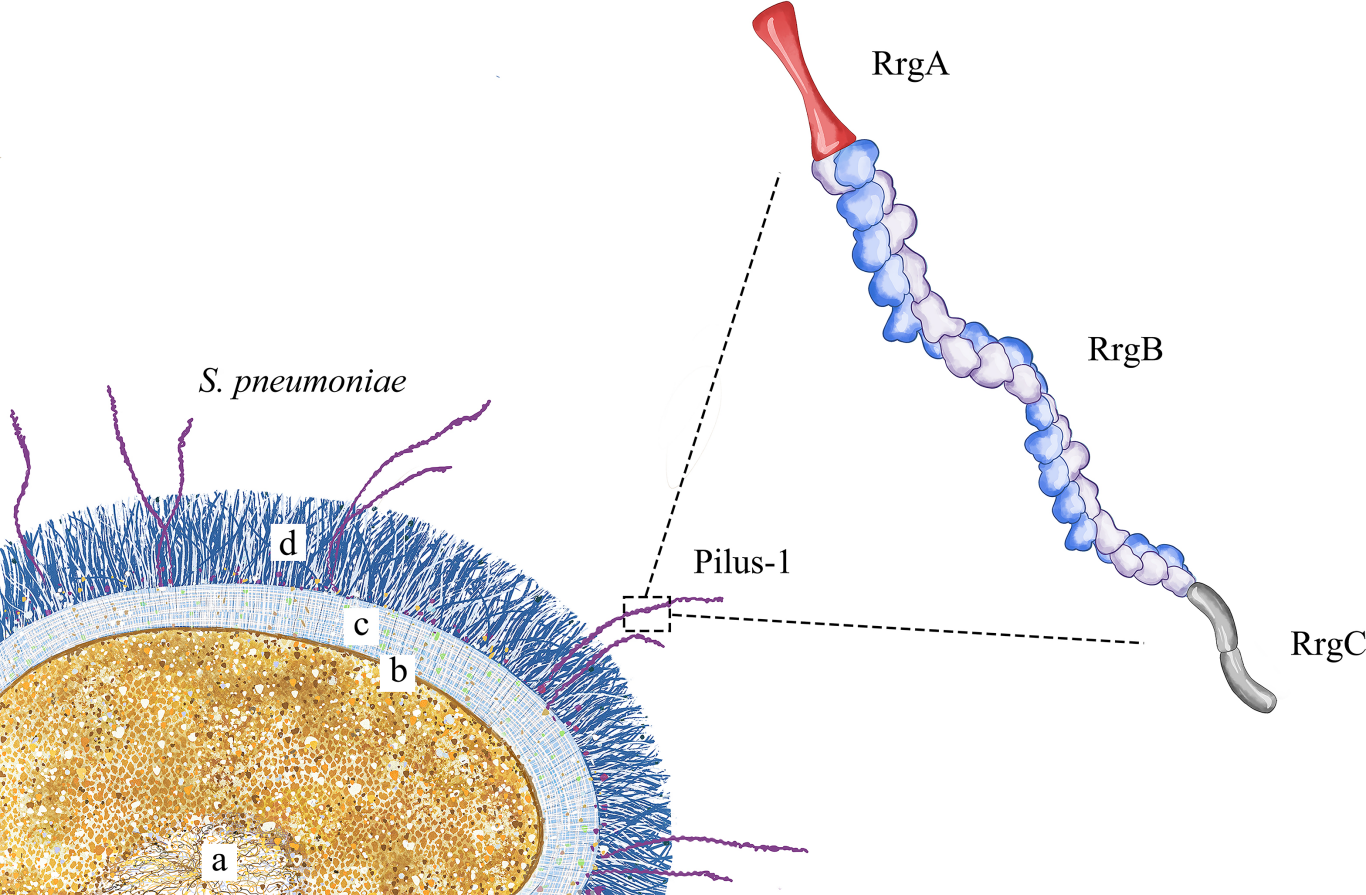
Pilus-1 schematic diagram. *S. pneumoniae* is shown in the lower left, where **(a)** is the nucleoid, **(b)** is the cell membrane, **(c)** is the cell wall, and **(d)** is the capsule. The enlarged structure on the right is pilus-1, including RrgA at the top, the backbone protein RrgB in the middle and RrgC at the bottom.

Pilus-1 (**Figure 1**) is present all over the surface of *S. pneumoniae*, and electron microscopy has shown that it has a diameter of approximately 2-6 nm (Falker et al., 2008) and a length of more than 1.5 μm. Pilus-1 is composed mainly of RrgA, RrgB and RrgC; a small amount of pilin RrgA is located at the top, and RrgC is at the bottom. RrgB is the backbone protein and plays a vital role in pilus-1 formation (Hilleringmann et al., 2009).

### 2.2 Structure of pilus-1

**Figure 2:**
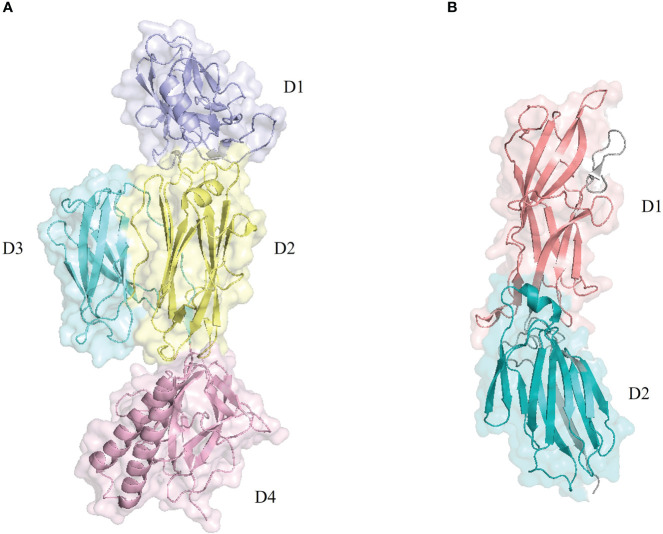
Pilus backbone protein structure diagram. The structures were retrieved from the RCSB Protein Data Bank and visualized with PyMOL. **(A)** The backbone protein RrgB (PDB: 3RPK) of pilus-1 includes four structural domains: domain 1 (D1) in purple, domain 2 (D2) in yellow, domain 3 (D3) in light blue, and domain 4 (D4) in light pink. **(B)** The backbone protein pitB (PDB: 7F7Y) of pilus-2 includes two structural domains: domain 1 (D1) in pink and domain 2 (D2) in blue.

RrgB (PDB ID: 3RPK) is composed of four domains (**Figure 2A**), with a nose-like bulge in spatial configuration and polarity. The protein subunits are connected head to tail to form protofilaments, and two protofilaments are intertwined to form the main structure of *S. pneumoniae* pilus-1 (Hilleringmann et al., 2008). Due to the sequence variability of PI-1, there are currently three isoforms (RrgB clade I, RrgB clade II, RrgB clade III), and the protein homology among the different isoforms ranges from 48% to 60%. The homology among the proteins is >99% (Moschioni et al., 2008), and the sequences of the same isoforms are highly conserved. The crystal structure of the C-terminal major part of RrgB (residues 184–627) has been solved at 2.8 Å resolution, revealing three independently folded protein domains (D2–D4). The N-terminal independently folded domain D1 rarely interacts with the remaining RrgB structure, suggesting that D1 may be more flexible than D2–D4 (Paterson and Baker, 2011). One study tested single recombinant RrgB domains D1–D4 in active and passive immunization, and the results showed that the functional domain D1 was the most effective, providing a level of protection comparable to that of the full-length protein (Gentile et al., 2011).

RrgA is the main pilus adhesin (Nelson et al., 2007). It is distributed primarily in the distal end of pilus-1, is arranged in clusters, and is the apical protein of pilus-1, mediating the adhesion function of the pilus and interacting with collagen, fibronectin and laminin. Similar to findings in other gram-positive bacteria, RrgA is not required for the initial formation of *S. pneumoniae* pilus-1. The RrgA protein contains four domains, including an integrin I collagen recognition domain consisting of two intervening “arms” folded into a positively charged cradle structure and three stem-forming domains (Shaik et al., 2014). Because the homology of clade I and clade III RrgAs encoded by PI-1 is >99%, only two isoforms of RrgA exist; the variation between the two isoforms is mainly concentrated in the protein head, while the slender stem is conserved (Moschioni et al., 2010b). RrgA has 893 residues, and its crystal structure reveals a 195 Å long elongated protein with four domains that compactly fold with a limited size. The binding of the main part of RrgA to RrgB occurs via an interaction catalyzed by the specific sortase at the C-terminal end of its D4 domain, and the four domains of RrgA rarely come in contact with each other, indicating potential flexibility (Izore et al., 2010).

RrgC is distributed mainly at the end of *S. pneumoniae* pilus-1 near the bacterial cell surface. It contains three domains connected by two isopeptide bonds, and the structure is curved and rod-like. During the polymerization of subunits, RrgC does not depend on the sortase encoded by PI-1; rather, this process is catalyzed by the housekeeping sortase SrtA, which anchors *S. pneumoniae* pilus-1 on the bacterial surface (Shaik et al., 2014). Unlike RrgA and RrgB, the RrgC protein subunit is highly homologous (>98%) among *S. pneumoniae* strains.

### 2.3 Characteristics of pilus-2

PI-2 encoding *S. pneumoniae* pilus-2 consists of five genes: the signal peptide-like protein gene *sipA*; *pitA* and *pitB*, which encode two surface structural proteins, and the sortase genes *srtG1* and *srtG2*. The entire islet is located between the protease T gene (*PepT*) and the ferrochelatase gene (*HemH*) (Bagnoli et al., 2008; Shaik et al., 2015). In recent years, the prevalence of *S. pneumoniae* pilus-2 has increased to approximately 20%, and it is mainly associated with serotypes 1, 2, 7F, 19A and 19F (Zahner et al., 2010; Hjalmarsdottir et al., 2015). Pilus-2 is composed predominantly of repeating units of the structural protein PitB, whose covalent polymerization is catalyzed by the homologous sortase SrtG1 and SipA. PitB contains two domains, D1 and D2, which are linked by SrtG1 through recognition of the VTPTG motif. PitA and SrtG2 are not necessary for pilus-2 (Bagnoli et al., 2008).

### 2.4 Structure of pilus-2

In contrast to pilus-1 of *S. pneumoniae*, whose complex structure has been well studied, pilus-2 is thus far poorly understood. The structure of PitB (PDB ID: 7F7Y), the backbone pilin, has been resolved to 2.8 Å. It is 110 Å long and 40 Å wide and consists of two domains, D1 and D2 (**Figure 2B**), whose covalent binding is catalyzed by the sortase SrtG1 (Yadav and Krishnan, 2022). The two domains of PitB present an irregular structure; the N-terminal D1 domain consists of 12 chains, and the C-terminal D2 domain consists of 11 chains (Shaik et al., 2015). The interaction between D1 and D2 is similar to that observed in RrgB (Paterson and Baker, 2011; El Mortaji et al., 2012). However, the structural protein PitA and the sortase SrtG2 do not play important roles in the formation of pilus-2.

## 3 Functions of *S. pneumoniae* pili and their correlations with serotypes

Although pili are not essential structures for the survival of *S. pneumoniae*, they play important roles in the initial stage of *S. pneumoniae* infection, forming a structural basis and acting as virulence factors that mediate bacterial adhesion to host cells. Studies have shown that pili play direct roles in promoting the adhesion, colonization and pathogenesis of *S. pneumoniae* (Barocchi et al., 2006; Kreikemeyer et al., 2011).

### 3.1 Biological functions of pili

Local invasive infection is the basis for bacteremia. In the context of respiratory infection with *S. pneumoniae*, in one study on the adhesion function of *S. pneumoniae* pilus-1, Nelson et al. (Nelson et al., 2007) used human alveolar epithelial A549 cells as host cells and found that although *rrgA*-knockout bacteria formed pili, their adhesion ability was significantly reduced. In contrast, the *rrgB-* and *rrgC*-knockout bacteria did not form pili, but their adhesion ability was similar to that of the wild-type bacteria with pili. These findings suggest that the adhesion function of *S. pneumoniae* pilus-1 is determined by RrgA rather than the pilus backbone protein. In a preliminary study on the immune mechanism of *S. pneumoniae* pilus-1, Nelson et al. found that compared with pilus-deficient strains, pilus-carrying strains not only had advantages in colonization, pathogenicity and septicemia induction but also effectively induced the release of tumor necrosis factor alpha (TNF-α) and interleukin 6 (IL-6) and enhanced the host’s inflammatory response. Subsequently, it was found that this proinflammatory effect of pilus-1 is mediated by the surface-exposed domain 3 of RrgA, particularly the 49-amino-acid sequence, which acts as a TLR2 receptor agonist (Basset et al., 2013). In a study on the *S. pneumoniae* pilus-1 protein subunit RrgA and the mechanism of host immune damage, Orrskog et al. (Orrskog et al., 2012) used *rrgA* knockout and nonknockout strains through intranasal and intraperitoneal challenge experiments and found that infection with strains expressing RrgA led to earlier development of sepsis and more rapid disease progression in wild-type mice than infection with complement receptor 3 (CR3) antibody and infection in CR3-deficient mice, suggesting that RrgA can influence macrophage function and systemic infection status by interacting with CR3. The uptake of *S. pneumoniae* by murine and human macrophages is enhanced for strains that express RrgA, and RrgA-CR3-mediated phagocytosis promotes the spread of localized to systemic infections. Basset et al. (Basset et al., 2013) showed that RrgA can enhance virulence and the inflammatory response by activating TLR2.

Infections other than respiratory system infections caused by *S. pneumoniae* mostly develop from bacteremia caused by respiratory tract invasion by pathogenic bacteria. *S. pneumoniae* is the main cause of bacterial meningitis (Iovino et al., 2016a). With regard to meningeal infection caused by *S. pneumoniae*, Iovino et al. (Iovino et al., 2016b) conducted an intravenous challenge experiment and found that the bacterial loads of the pilus-carrying strains were higher in the brain; when *rrgA* was knocked out, the load of the pilus-carrying strains in the brain decreased. Additionally, strains that expressed RrgA more readily dispersed to form a single coccus, and based on findings by high-resolution immunofluorescence microscopy, these strains were more likely to adhere to the vascular endothelium of the blood−brain barrier, promoting the passage of bacteria through the blood−brain barrier. This finding suggests that the adhesin RrgA of pilus-1, as a virulence factor associated with *S. pneumoniae* meningitis, plays an important role in promoting the adhesion of individual *S. pneumoniae* to the vascular endothelium of the blood−brain barrier. Further research on adhesion ligands (Iovino et al., 2017) has shown that RrgA can bind to polymeric immunoglobulin receptor (pIgR) and platelet endothelial cell adhesion molecule (PECAM-1) on the surface of blood−brain barrier epithelial cells and that downregulation of these two receptors has the potential to prevent *S. pneumoniae* meningitis. Blocking the receptors pIgR and PECAM-1 or RrgA in the pilus significantly reduces brain invasion by *S. pneumoniae* and increases mouse survival (Iovino et al., 2018). These findings offer promising prospects for new treatments.

To investigate middle ear infections caused by *S. pneumoniae*, Figueira et al. (Figueira et al., 2014) created a middle ear infection model by nasal challenge and analyzed the middle ear fluid. The results showed that although *S. pneumoniae* pilus-1 was nonessential for *S. pneumoniae* infection of the middle ear, the bacterial loads were higher for pilus-carrying strains than for pilus-deficient strains, suggesting that *S. pneumoniae* pilus-1 promotes middle ear infections. The results of the above studies demonstrate that *S. pneumoniae* pilus-1 plays a vital role in bacterial adhesion, invasion, host immune damage, and metastasis of infection foci and that the RrgA protein subunit is the main adhesion factor and immune mediator.

All the above studies have shown that although the skeletal protein of *S. pneumoniae* pilus-1 is RrgB, the main functional protein is RrgA. *S. pneumoniae* pilus-1 has adhesion functions and virulence effects, exacerbates inflammatory damage in the host and is vital in the local and systemic immune response.

In contrast to *S. pneumoniae* pilus-1, Bagnoli et al. (Bagnoli et al., 2008) found, through an adhesion experiment with A549 cells, that pilus-2 aids in adhesion through the backbone protein PitB and that the adhesion ability is weaker than that of RrgA. Collagen, fibronectin and laminin are the common adhesion ligands of pilus-1 and pilus-2.

### 3.2 Relationships between pilus and vaccine serotypes

The relationships between the epidemiology of *S. pneumoniae* pili and the serotypes covered by vaccines have received extensive attention. Kawaguchiya et al. found that 19F, 23F and 19A were vaccine-covered serotypes among the isolates carrying PI-1, while 6E, 15B and 35B were nonvaccine types, and that pneumococcal conjugate vaccine (PCV) covered most of the pilus-1-carrying strains (Kawaguchiya et al., 2017). Pilus-1 is not widely distributed among the 100 known *S. pneumoniae* serotypes (Ganaie et al., 2020); analyses of global isolates have shown that the overall frequency of pilus-1 is 30%, suggesting that the islet is associated with pneumonia. There is a certain correlation among the genotypes of *S. pneumoniae* isolates (Aguiar et al., 2008; Moschioni et al., 2008). Aguiar et al. (Aguiar et al., 2008) found an association between 83% of *rlrA*-positive isolates and vaccine serotypes 4, 6B, 9V, and 14 (all covered by PCV7) in their study of the pilus regulation gene *rlrA*; in other studies, this gene has been found to be absent in nonvaccine serotypes (1, 7F, 8 and 12B). Among the 305 *S. pneumoniae* isolates collected worldwide, pilus-2 has been found in 16.4%. Most pilus-2 strains are associated with serotypes 1, 2, 7F, 19A, and 19F (all vaccine-covered serotypes) (Bagnoli et al., 2008). Serotypes 7F and 19A are the serotypes that most frequently carry PI-2, followed by 1 and 19F (Zahner et al., 2010). A study from Iceland showed that among 398 isolates, 88.1% (118 isolates) and 81.6% (31 isolates) of PI-1-positive and PI-2-positive isolates, respectively, belonged to vaccine serotypes. PI-1-positive isolates were most common in serotypes 19F and 6B, and PI-2-positive isolates were most common in serotype 19F.

Overall, *S. pneumoniae* pili are generally associated with serotypes covered by PCVs. However, in the long term, the ongoing phenomenon of serotype replacement with vaccine introduction (Lo et al., 2022) means that pilus isolates could also be replaced. One study reported a prevalence of 23.7% for pilus-1 isolates in 2001, one year after the introduction of PCV7 in the United States, and 14.9% in 2004. However, by 2007, the prevalence of nonvaccine pilus isolates had increased to 26.1% (Regev-Yochay et al., 2010). The analysis showed that the decline in the prevalence of pilus-1 among *S. pneumoniae* isolates from 2001 to 2004 was due primarily to the replacement of vaccine-type (VT) strains with nonvaccine-type (NVT) strains. However, between 2004 and 2007, the increase in prevalence was due mainly to the increased frequency of pili in NVT strains. The results reported in this review indicate that the application of PCVs will have an impact on the emergence of new cloned strains and provide new ideas for future *S. pneumoniae* pathogenic surveillance.

## 4 Potential of *S. pneumoniae* pilins as vaccine candidates

### 4.1 Limitations of traditional vaccines

At present, the traditional pneumococcal polysaccharide vaccine (PPV) and PCV are widely used in many countries and regions. Due to the immaturity of the immune systems of young children, the polysaccharide antigens used in PPV cannot produce ideal protective efficacy in infants under 2 years of age (Kadioglu et al., 2008). This limitation prompted the creation of the pneumococcal 7-valent conjugate vaccine (PCV7), which covers serotypes 4, 6B, 9V, 14, 18C, 19F, and 23F. In addition, PCV10 and PCV13 have also been included in immunization programs in more than 120 countries (Ubukata et al., 2015). Although PCV can elicit an effective immune response, it has limited serotype coverage and is expensive to manufacture. It has been reported (Regev-Yochay et al., 2010; Regev-Yochay et al., 2016) that after planned immunization with PCV7, the prevalence of *S. pneumoniae* in the population does not change significantly, but the proportion of nonvaccine strains among *S. pneumoniae* epidemic strains gradually increases. This result suggests that after the large-scale use of serotype-dependent vaccines, serotype replacement occurs in the *S. pneumoniae* epidemic strain, which reduces the protective efficacy of the vaccines.

### 4.2 Application prospects of pilin

Many studies have shown that a variety of *S. pneumoniae* virulence factors can be used as candidate antigens for novel protein vaccines (Kim et al., 2017), providing broad-spectrum protection against *S. pneumoniae* infection. These include, for example, choline-binding proteins (CBPs) (Bittaye and Cash, 2015), pneumoniae surface proteins A and C (PspA/PspC) (Tostes et al., 2017), heat shock proteins (Hsp40/DnaJ) (Cui et al., 2011), pneumococcal surface adhesin (PsaA) (Converso et al., 2017) and pneumolysin (Ply) (Brooks et al., 2015). However, there is currently no commercialized protein vaccine that can protect against *S. pneumoniae* infection. Continued discovery of new protein antigenic sites may not only effectively promote the development of protein vaccines with better protective efficacy but also facilitate the identification of drug targets through the study of immune protection mechanisms.

Studies (Moschioni et al., 2010a; Regev-Yochay et al., 2010) have found that serotypes and *S. pneumoniae* pili are correlated. In the initial stage after traditional *S. pneumoniae* vaccine immunization, the expression of pilus-1 in epidemic strains decreased sharply in a short period due to the rapid reductions in vaccine-type strains; however, the expression has increased yearly, even exceeding the level before the popularization of traditional vaccines, especially in vaccine-type strains and drug-resistant strains. This finding indicates that the *S. pneumoniae* pilus islet has mobile genetic elements and that pilus-1 plays an important role in the pathogenicity and resistance of epidemic strains. Pilus-1 can thus be used as a candidate antigen target for the development of protein vaccines and further in-depth research. In addition, there is a strong link between pili and antimicrobial resistance, and the above findings could lay a foundation for using pili as potential antigens for future protein vaccines.

### 4.3 Status of pilin research

Early developed commercial *S. pneumoniae* vaccines used capsular polysaccharide (CPS) as the antigen (PPV23), aiming to reduce *S. pneumoniae* infection by inhibiting the colonization of specific serotype strains (Duke and Avci, 2023). To improve the defect of poor immunogenicity, conjugation technology was introduced to covalently combine CPS with immunogenic carrier proteins to develop glycoconjugate vaccines (Avci, 2013; Avci et al., 2019). Multiple CPS-protein conjugate vaccines including V116 (Platt et al., 2023), VAX24 (Fairman et al., 2021) and PNEUMOSIL (Duke and Avci, 2023) have been approved to enter clinical trials. Although the majority of invasive pneumococcal disease (IPD) strains are encapsulated organisms with typed serotypes, nontypeable isolates (including nonencapsulated isolates) can also cause IPD (Scott et al., 2012). Nonencapsulated strains are easily transformable due to the lack of capsules (Pearce et al., 2002) and adhere well to the respiratory epithelial cells, which has advantages in nasopharyngeal colonization, further exacerbating the limitations of CPS-based vaccine applications. As an alternative or supplementary strategy to conjugate vaccines (PCVs), pneumococcal protein vaccines aim to overcome serotype dependence and promisingly provide broad-spectrum protection at a relatively low cost, providing an option for children in developing countries.

Preliminary research on *S. pneumoniae* pilin vaccines has focused mainly on *S. pneumoniae* pilus-1, which has filamentous structures and can be effectively identified by the host immune system. Gianfaldoni et al. (Gianfaldoni et al., 2007) used recombinant pilus-1 protein for the first time to evaluate the protective efficacy of different proteins against *S. pneumoniae* in mice and found that RrgA, RrgB and RrgC stimulate the production of specific antibodies. Among them, RrgA and RrgB have immunoprotective efficacy that can significantly prolong the survival time of mice challenged by a lethal *S. pneumoniae* dose; notably, the protective efficacy of RrgC immunization was poor. Further studies by Moschioni et al. (Moschioni et al., 2010b) and Harfouche et al. (Harfouche et al., 2012) have found that there is cross-immunity between the two isoforms of RrgA and that immunization with RrgA clade II antigens can protect mice from lethal-dose challenge with the RrgA clade I strain. This result indicates that RrgA is not only immunogenic but also well conserved, making it an ideal candidate protein for pilus vaccines. However, there are no cross-protective effects among the three isoforms of RrgB. To address this problem, researchers have used the *rrgB* of *S. pneumoniae* TIGR4 (RrgB clade I), *S. pneumoniae* 6BSPEC (RrgB clade II), and *S. pneumoniae* 35BSME15 (RrgB clade III) to construct a fusion protein, RrgB321, containing three RrgB isoforms. The subunits of the different isoforms are connected by Gly-Ser-Gly-Gly-Gly-Gly-Gly so that the fusion protein can fold normally. The newly constructed fusion protein antigen RrgB321 exhibits good protective efficacy against *S. pneumoniae* in mice after active and passive immunization. In addition, the level of complement-dependent bacterial phagocytosis in pilus strains is comparable to that observed with PCV7, indicating that the RrgB321 component vaccine leads to successful immune protection against *S. pneumoniae* with pilus-1, covering more than 30% of the total *S. pneumoniae* strains. Unlike RrgB321, a single fusion protein component, PnuBioVax developed by Entwisle et al. is a whole-cell vaccine produced from genetically modified *S. pneumoniae* TIGR4 (Entwisle et al., 2017). Its enriched surface-exposed antigens include but are not limited to pilins RrgB and RrgA, pneumococcal surface adhesin A (PsaA) and non-toxic pneumolysin (Ply). Antibody responses to the TIGR4-type pilins RrgB and RrgA in subjects were shown to increase by more than 2-fold.

An RrgB fusion protein vaccine developed by Novartis is in preclinical trials (Harfouche et al., 2012). Intranasal mucosal immunization can reduce bacterial load in the early middle ear fluid of mice with experimental *S. pneumoniae*-related otitis media. However, due to limited antigenic targets, the pilin vaccine provides immune protection only against pilus-carrying strains, not against pilus-deficient strains. In addition, in the presence of pilin antibodies, the bacteria can selectively not express pili to evade immune attack. To overcome the limitations of pilins and enhance the immune coverage of vaccines, multivalent vaccines can be prepared in combination with bacterially conserved proteins. For example, a triple-protein vaccine consisting of pneumococcal choline-binding protein A (PcpA), pneumococcal histidine trimer protein D (PhtD), and detoxified pneumolysin (dPly) and a triple-protein vaccine composed of *Haemophilus influenzae* protein D, dPly, and PhtD have entered clinical phase II trials (Feldman and Anderson, 2014). As important components of protein-based vaccines, dPly, PcpA and PhtD participate in the compatibility of various protein vaccines (Brooks et al., 2015; Odutola et al., 2017; Odutola et al., 2019) and have entered clinical trials. Pili, with strong immunogenicity, are important for pathogenicity and induction of protective immunity against *S. pneumoniae* (Mora et al., 2005; Ahmed et al., 2014). Combining pilins with mature protein components is promising to exploit the dual advantages of easy recognition by the immune system and high conservation of thalline proteins. Furthermore, the study by Narciso et al. used the highly conserved lipoproteins MalX and PrsA in membrane particles (MPs) as antigens and provided us with new ideas (Narciso et al., 2022). MPs could be used as a platform for the preparation of protein-based vaccines against pneumococcal infections, with MalX and PrsA as the main antigens responsible for cross-protection. Membrane vesicles can promote immune responses and contribute to the transportation of virulence factors, and are promising for use as vaccine adjuvants and carriers (Jan, 2017; Malekan et al., 2020).

In addition to the licensed carrier proteins tetanus toxoid (TT), diphtheria toxoid (DT), CRM_197_, Haemophilus protein D (PD), etc., researchers have begun to pay attention to the impact of different types of carriers on the protective efficacy of vaccines, including outer membrane vesicles (OMVs) and generalized modules for membrane antigens (GMMA), virus-like particles (VLP) and nanomaterials, etc. (Gong et al., 2022; van der Put et al., 2023). Different immunization doses, immunization routes and adjuvants can also strongly affect the effects of pilin vaccines. Adjusting the protein compatibility ratio between different antigens of a combined vaccine, trying various immunization routes, and selecting appropriate immune adjuvants can help to optimize the protective efficacy provided by a pilin vaccine (Principi and Esposito, 2018).

## 5 Discussion

*S. pneumoniae* pili mediate bacterial adhesion, tissue colonization, and barrier invasion; induce inflammatory damage in the body; and play a vital role in the infection process. Research on the structures and functions of pili has gradually expanded, but the protective efficacy of pili as protein vaccine candidates and the mechanisms of their effects need to be further studied. After the popularization of PPV and PCV, expectations are high for *S. pneumoniae* vaccines in the face of changing epidemic strains and the rebound of pilus expression. Protein vaccines have become a hot topic in the field of *S. pneumoniae* vaccine research due to their low cost, strong conservation, and lack of serotype-specific restrictions. Although current research on protein vaccines involves multiple levels, there is no ideal protein vaccine on the market yet. It is expected that universal protein vaccines with broad-spectrum immune protection will be developed. Pili not only play important roles in the pathogenic process of *S. pneumoniae* but also meet the selection criteria for vaccine candidate proteins in terms of their biological functions, immunogenicity, extracellular expression, and induction of antibody production. They thus have great potential for use in vaccine research and development. Current research on recombinant *S. pneumoniae* pilin vaccines is still relatively limited. Continued discovery of new protein antigen targets and evaluation of protein vaccine combination modes remain promising research directions for universal *S. pneumoniae* protein vaccines.

## Author contributions

CM: Visualization, Writing – original draft, Software. YC: Writing – original draft, Supervision. ZY: Writing – original draft, Visualization. YJ: Supervision, Writing – review & editing, Funding acquisition.
